# Higher frequency of subclonal anti-EGFR resistance mutations in post-treatment samples from patients with colorectal cancer liver metastases following anti-EGFR-based conversion chemotherapy

**DOI:** 10.1371/journal.pone.0351243

**Published:** 2026-07-06

**Authors:** Christoph Steup, Antonia Mondorf, Peter Wild, Ursula Pession, Wolf Otto Bechstein, Florian A. Michael, Melanie Winter, Julia Bein, Stefan Zeuzem, Jörg Trojan, Christine Koch

**Affiliations:** 1 Goethe University Frankfurt, University Hospital Frankfurt, Department of Internal Medicine, Frankfurt am Main, Germany; 2 Goethe University Frankfurt, Dr. Senckenberg Institutes of Pathology and Human Genetics, University Hospital Frankfurt, Frankfurt am Main, Germany; 3 Goethe University Frankfurt, University Hospital Frankfurt, Department of General, Visceral, Transplant and Thoracic Surgery, Frankfurt am Main, Germany; Dana-Farber Cancer Institute, Harvard Medical School and THE Broad Institute of MIT and Harvard, LEBANON

## Abstract

**Background/aims:**

Initially unresectable colorectal liver metastases may become resectable after chemotherapy. The acquisition of somatic mutations has been demonstrated to indicate evolving tumor subclones resistant to therapy and hinder R0 resection. However, the added value of mutational profiling in a real-world setting remains to be elucidated. The objective of the present study was to identify recurrent molecular profiles in colorectal cancer liver metastases that had been pre-treated with anti-EGFR monoclonal antibodies plus chemotherapy.

**Methods:**

A retrospective, single-centre analysis of colorectal cancer liver metastases samples from 30 patients (9 pre-therapy/21 post-therapy) who received chemotherapy and anti-EGFR agents between January 2008 and February 2014 was conducted. Targeted next-generation resequencing (NGS) was performed. Subsequently, the results were compared to publicly available genomic datasets of primary colorectal cancer and colorectal cancer liver metastasis.

**Results:**

145 mutations were identified (mean, 5.2 ± 1.3 mutations per sample; range 0–28, median 2, IQR 3.75). Neither mutation count nor RAS status did correlate with the achievement of resectability. NGS confirmed prior identified KRAS mutations in 4/29 patients (14.8%) and revealed further somatic RAS alterations in 5 cases. In addition to the classical colorectal cancer driver mutations, tumors exhibited recurrent mutations in genes implicated in anti-EGFR resistance such as HNF1A (34.4%, 10/29), FGFR2 (31%, 9/29), VHL (27.5%, 8/29) and PDGFR (27.5%, 8/29), ERBB2 (24.1%, 7/29), ABL1 (24.1%, 7/29), SMO (24.1%, 7/29), GNA11 (20.6%, 6/29), RET (20.6%, 6/29), STK11 (20.6%, 6/29), HRAS (20.6%, 6/29) and NRAS (17.2%, 5/29).

**Conclusion:**

In the current study, we describe a higher frequency of potential anti-EGFR treatment resistance mutations in patients who respond well to the combination of chemotherapy and anti-EGFR monoclonal antibody treatment. Nevertheless, subclonal mutations associated with resistance to anti-EGFR therapy did not affect the secondary resectability of colorectal liver metastases.

## Introduction

Despite advances in targeted and combinatorial treatment approaches, colorectal cancer (CRC) remains a leading cause of cancer-related death [1,2]. Approximately 50% of patients develop metastatic colorectal cancer (mCRC) during the course of the disease, with a median overall survival (mOS) of approximately 30 months [3]. In mCRC patients, a combination of cytotoxic chemotherapy and targeted therapy remains the treatment backbone [4–6]. The mortality associated with CRC is related to the development of metastases^7^ and, later, duration of treatment response is affected by secondary treatment resistance by acquired drug resistance, particularly to targeted therapies such as anti-epidermal growth factor receptor (anti-EGFR) monoclonal antibodies [7–10].

Activation of EGFR primarily signals through the mitogen-activated protein kinase (MAPK) pathway, regulating cellular proliferation via the RAS–RAF–MEK–ERK cascade [11], but it also engages parallel signaling pathways such as the PI3K–AKT pathway, which contributes to cell survival and resistance mechanisms [12].

Most studies have indicated that resistance to anti-EGFR therapy is predominantly associated with mutations affecting components of the MAPK pathway, particularly in *KRAS, NRAS,* and *BRAF* [13]. However, it should be noted that many of these findings have been derived from analyses of initial responders who developed secondary disease progression under anti-EGFR monotherapy without concomitant chemotherapy [14,15]. In addition to direct alterations within the MAPK pathway, resistance can arise through activation of bypass signaling pathways that restore downstream MAPK signaling despite EGFR inhibition. These include upstream activation of alternative receptor tyrosine kinases such as ERBB2, MET, STAT3, YAP and FGFR, as well as ligand-mediated activation of parallel pathways [16]. Furthermore, alterations in the PI3K–AKT pathway and other compensatory signaling networks may contribute to resistance by promoting cell survival independently of EGFR blockade [17]. Together, these well-established resistance pathways highlight the complexity of resistance biology and underscore the need for comprehensive molecular profiling.

Currently, two anti-EGFR monoclonal antibodies are approved for clinical use in metastatic colorectal cancer (mCRC): the chimeric IgG1 monoclonal antibody cetuximab and the fully human IgG2 monoclonal antibody panitumumab. Both agents bind to the extracellular domain of the epidermal growth factor receptor (EGFR), thereby preventing ligand binding, receptor dimerization, and downstream signaling activation [18].

Anti-EGFR antibodies have demonstrated clinical efficacy across different treatment lines in mCRC, either as monotherapy or in combination with cytotoxic chemotherapy [18]. Analyses of major randomized clinical trials have consistently shown that their benefit is largely restricted to patients with left-sided primary tumors and tumors lacking activating alterations in the RAS signaling pathway [19]. In the presence of RAS mutations, constitutive downstream pathway activation renders EGFR inhibition ineffective, thereby explaining primary resistance in these patients.

Accordingly, current international guidelines [4,5]recommend anti-EGFR antibodies in combination with a doublet chemotherapy backbone as a preferred first-line treatment option for patients with left-sided, RAS wild-type mCRC, or in second line if they have been indicated in the first line but have not been used yet.

More recently, data from the PARADIGM study [20] have suggested that molecular “negative hyperselection” using circulating tumor DNA (ctDNA) to exclude anti-EGFR resistance-associated alterations may further refine patient selection [21]. This strategy may help identify a subset of patients with right-sided tumors who could potentially benefit from anti-EGFR therapy; however, this approach has not yet been established as a clinical standard.

Current commonly available mutational profiles from tumor tissues, obtained through either resection or biopsy, are usually biased towards untreated and mostly primary CRC. In pretreated patients, profiles are usually taken after progressive disease has become clinically apparent, with the emergence of one or more therapy-resistant clones. This is due to the need for sufficient, high-quality tumor material for next-generation sequencing (NGS)-based analysis, and to avoid unnecessary invasive biopsies without clinical implications [22,23]. Therefore, especially subclonal mutations fostering partial resistance to therapy in well responding patients and thereby limiting further tumor shrinkage in order to achieve resectability of colorectal cancer liver metastasis are underrepresented in the majority of previously published datasets. In line, sequential analysis of cell-free tumor DNA (cfDNA) has indicated subclonal mutations in the epidermal growth factor receptor (EGFR), which most likely occur as a response to selective anticancer treatment pressure from anti-EGFR directed medications [15]. On average, five resistance mutations bypassing the need of EGFR signaling were found in cfDNA analysis. Nevertheless, analysis of cfDNA is skewed towards dominant clonal populations and therefore underrepresents the low number of surviving remaining cancer cells after successful pretreatment of colorectal cancer liver metastasis before resection. Due to their low tumor burden and functionally inactive state, they often lack cfDNA shedding [24].

Little is known about mutational patterns in patients with colorectal cancer liver metastasis after anti-EGFR plus chemotherapy pretreatment. Therefore, we performed a targeted panel-based NGS of several colorectal cancer liver metastasis samples, pretreated with chemotherapy plus anti-EGFR therapy.

## Results

**Fig 1:**
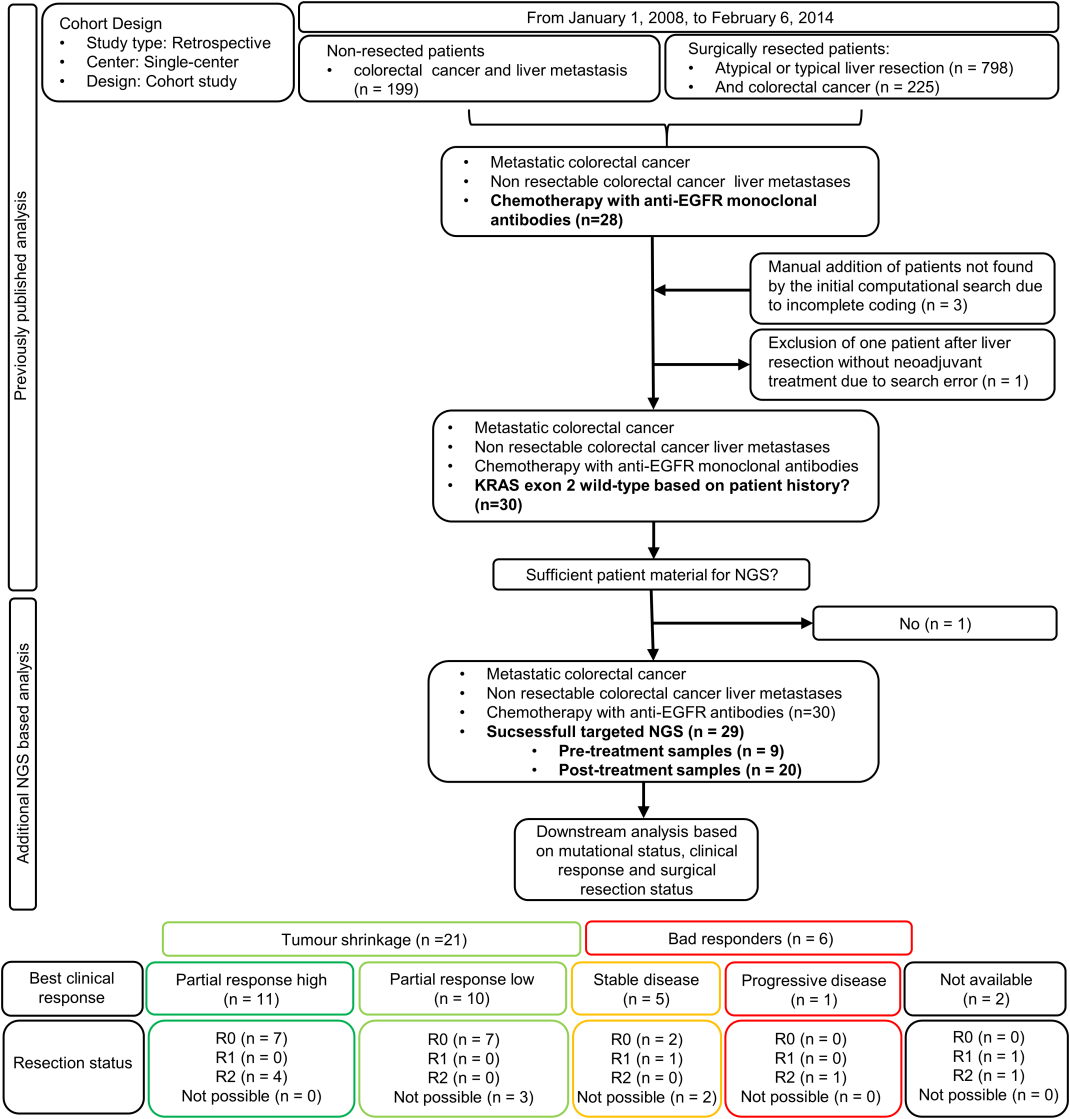
Consort Diagram and Patient Flow: Study design, patient selection, baseline characteristics, and reasons for exclusion are illustrated. Treatment response to systemic therapy consisting of anti-EGFR antibodies in combination with chemotherapy is shown and categorized as tumor shrinkage (partial response – high, partial response – low) or poor response (stable disease, progressive disease). Surgical resection status for colorectal cancer liver metastasis following systemic therapy are additionally depicted. Abbreviations: KRAS, Kirsten rat sarcoma viral oncogene homolog; NGS, next-generation sequencing.

Targeted next generation sequencing (NGS) was successfully conducted in 29 out of 30 previously published [25] colorectal cancer liver metastases samples (Fig 1, The list of panel gene targets can be found in the methods section). 9/29 (31%) and 20/29 (69%) of samples were collected pre-treatment/untreated and post-treatment, respectively. Treated patients received chemotherapy plus anti-EGFR agents in a neoadjuvant treatment approach between January 2008 and February 2014 before RAS mutations other than KRAS were identified as a predictive factor for anti-EGFR treatment (see Table 1 for a summary of previously published patient demographics and treatments). Median time from the start of systemic treatment to resection, was 7.6 months (range 4.06–11.15 months). 19.9% and 76.7% had right and left sided primary tumors, respectively.

**Table 1:** Basic Patient Characteristics.

Number of patients	29
Gender, male/female, n (%)	19 (63.3%)/11 (36.7%)
Age, years (m/f)	58.1/52.04
Synchronous/metachronous metastasis, n (%)	22/8 (73.3%/26.7%)
Neoadjuvant treatment in first line, n (%)	FOLFOX/cetuximab 14 (46.7%)
	FOLFIRI/cetuximab 11 (40%)
	FOLFIRI/panitumumab 1 (3.3%)
	FOLFOX/panitumumab 3 (10%)
	5-FU/cetuximab 1 (3.3%)
Localization Frequency, n (%)	Caecum 1 (3.3%)
	C. ascendens 4 (13.3%)
	C. transversum 1 (3.3%)
	C. sigmoideum 15 (50.0%) Rectum 8 (26.7%)
	Colon (unspecified) 1 (3.3%)

*Patient characteristics based on standard clinical criteria illustrated in absolute numbers and percent of included samples.*

*Abbreviations: C. = Colon, FOLFOX = chemotherapy regimen containing folinic acid, fluorouracil and oxaliplatin, FOLFIRI = chemotherapy regimen containing folinic acid, fluorouracil and irinotecan hydrochlorid*

**Fig 2:**
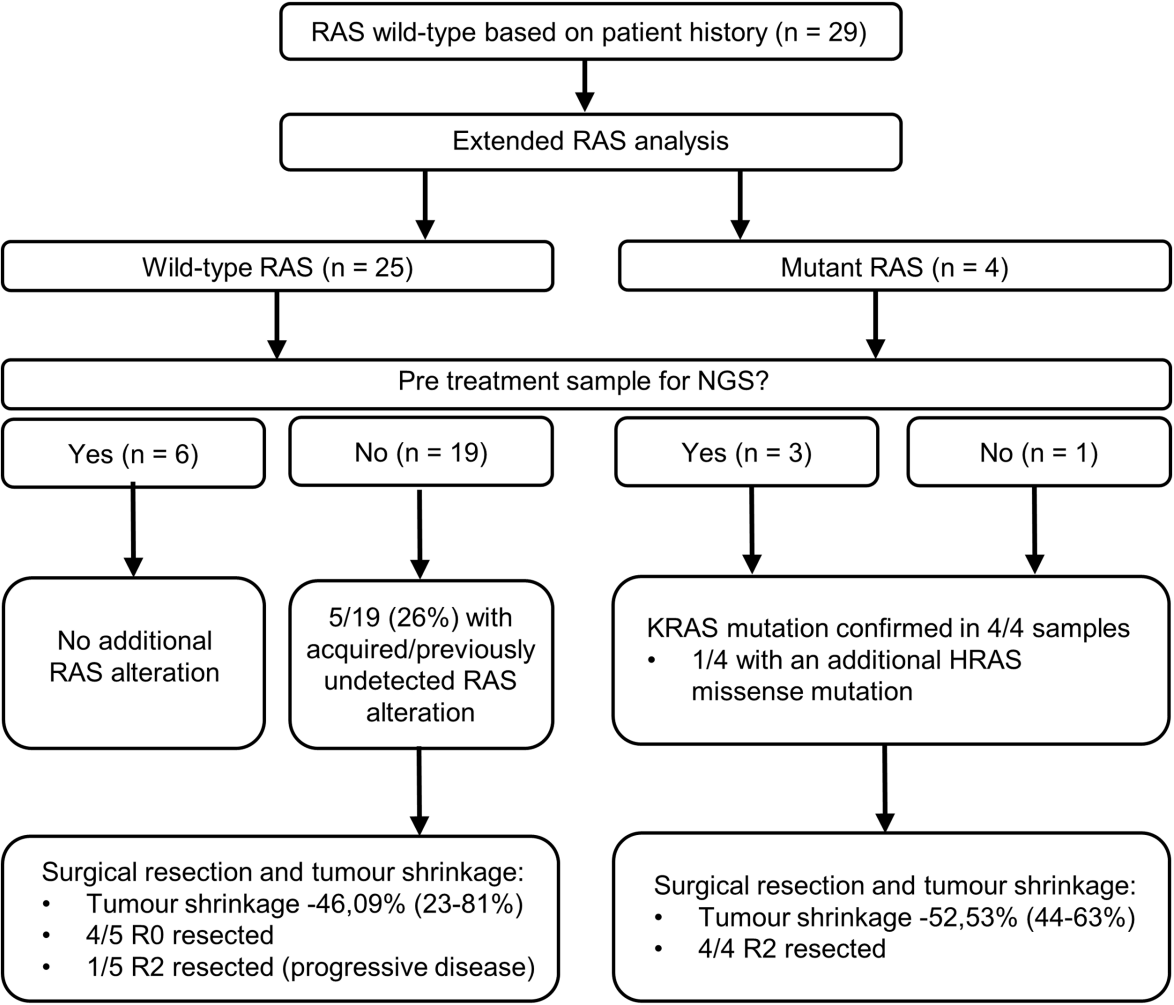
Patient RAS Status. RAS status was evaluated at three distinct time points.The first status was based on patient history, based on different modes of analysis. The second extended RAS analysis was performed within the previously published study [25] in a standardized approach. The last analysis was based on the results of targeted NGS presented in this study with an allel frequency >3%. Abbreviations: Rat sarcoma = RAS, KRAS = Kirsten rat sarcoma, HRAS = Harvey rat sarcoma, NGS = Next generation sequencing.

A total of 145 mutations were identified (mean, 5.2 ± 1.3 mutations per sample; range, 0–28; median, 2; IQR, 3.75). For the purpose of this analysis, only mutations with a variant allele frequency (VAF) greater than 3% were considered, ensuring that subclonal variants included in the study were reliably detectable. Mutation count did not correlate with the achievement of resectability (data not shown). NGS confirmed prior identified KRAS mutations in 4/29 patients (14.8%) and revealed further somatic alterations in RAS in 5/19 post-treatment samples. Surprisingly, post treatment/acquired RAS alterations did not influence successful R0 resection (4/5, 80%, all RAS mutant patients 4/9, 44.4%) or tumor shrinkage (Fig 2) in this highly preselected cohort, although none of the pretreatment KRAS mutated patients achieved R0 resection.

**Fig 3:**
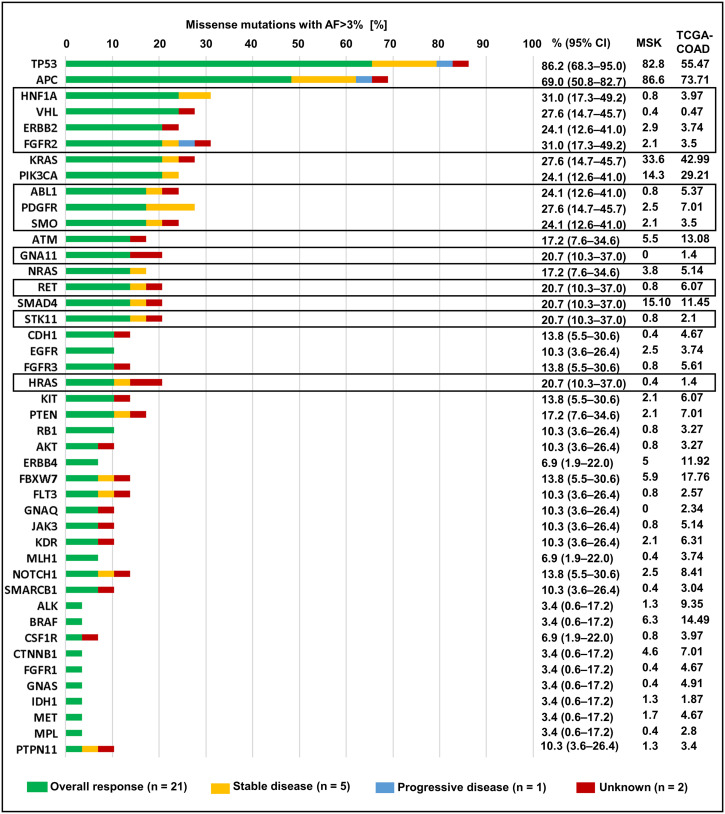
Missense Mutations in Colorectal Cancer Liver Metastasis. Panel-based NGS was performed. Samples with at least one missense mutation with an allele frequency (AF) >3% were counted and are displayed as a percentage of total samples (n = 29). Frequencies are compared to publicly available MSK-IMPACT Clinical Sequencing Cohort (MSK, n = 428, only colorectal cancer liver metastasis samples by filter), and The Cancer Genome Atlas- Colon Adenocarcinoma cohort (TCGA-COAD n = 461, mostly primary tumors (23% of samples without reported sampling side). For abbreviations of genes please see the table in the methods section.

**Fig 4:**
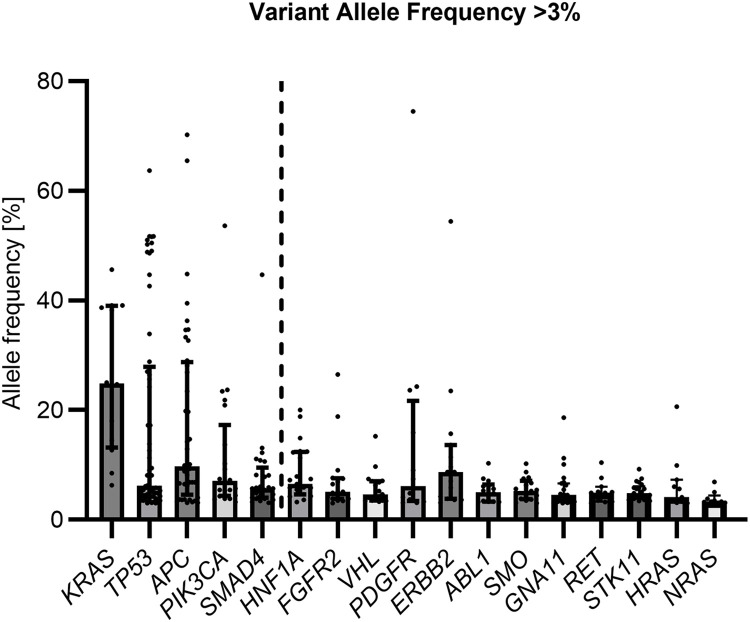
Variant Allele Frequency of Frequently Altered Genes. Bars are indicating the median of allele frequency for all samples with allele frequency above 3% (technical threshold), with error bars indicating interquartile range (IQR). For full gene names, please refer to the table in the methods section. Left of the vertical line are the well described clonal colorectal cancer driver mutations, without relevant differences to public databases. Right of the vertical line are genes which were detected in a higher frequency in SNVs in our dataset in contrast to public databases.

We further analyzed the publicly available The Cancer Genome Atlas (TCGA) – Colon Adenocarcinoma (COAD) cohort of the TCGA Pan-Cancer Atlas, which mostly consists of primary CRC samples and the liver metastasis samples from the MSK- Metastastic colorectal cancer cohort and compared mutation frequencies to our study. Somatic mutations (for a complete list of gene abbreviations and their full names, please refer to the table in the Methods section) in *APC* and *TP53* were identified in 68.9% (20/29) and 86.2% (25/29) of cases, respectively (Fig 3). Commonly mutated genes such as *PIK3CA* (24.1%, 7/29), *SMAD4* (20.6%, 6/29), *ATM* (17.2%, 5/29) and *FBXW7* (13.7%, 4/29) did not significantly differ in frequency between our cohort and publicly available data (Fig 3). Intriguingly, we found a higher frequency of clonal and subclonal mutations in *HNF1A* (34.4%, 10/29), *FGFR2* (31%, 9/29), *VHL* (27.5%, 8/29), *PDGFR* (27.5%, 8/29), *ERBB2* (24.1%, 7/29), *ABL1* (24.1%, 7/29), *SMO* (24.1%, 7/29), *GNA11* (20.6%, 6/29), *RET* (20.6%, 6/29), *STK11* (20.6%, 6/29), *HRAS* (20.6%, 6/29) and *NRAS* (17.2%, 5/29), especially in comparison to the mutational profile of colorectal cancer liver metastases (Fig 3 and Fig 4).

**Fig 5:**
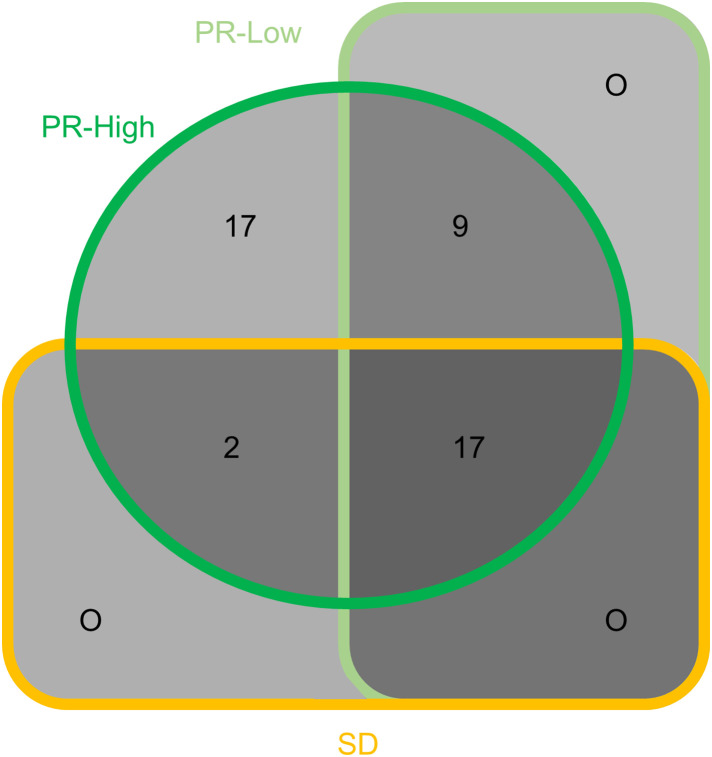
Venn Diagram: Mutations in Different Treatment Response Groups. Mutations were grouped based on their presence in the different treatment response groups and overlap is displayed in form of a venn diagram. Gene names according to the numbers are provided in Table 4. Abbreviations: PR-High = Partial Response-High (>50% tumor shrinkage according to RECIST), PR-Low = Partial Response-Low (>30% < 50% tumor shrinkage according to RECIST), SD = Stable Disease.

Next, we investigated if some of these more frequently observed mutations showed differential expression depending on therapy response (patients with a “partial response high” (PR-high, more than 50% tumor shrinkage according to RECIST) or “partial response low” (PR-low, 30–50% tumor shrinkage) compared to non-responders (stable disease (SD) and progressive disease (PD)) in patients with available sequential CT scans for response evaluation (27/29). While *HNF1A, FGFR2, PDGFR, ABL1, SMO, RET* and *STK11* did not show a difference in mutation frequency between responders and non-responders (Table 2), *VHL* (+33% in responders, 7/21 vs. 0/6), *ERBB2* (+28.6% in responders, 6/21 vs. 0/6) and GNA11 (+19% in responders, 4/21 vs. 0/6) showed mutations exclusively in responding patients. Successful R0 resection was performed in 14/21 (66,6%) patients with overall therapy response versus 2/6 (33,3%) in non-responders. Further, we analysed if the well-responding patients shared unique mutations not present in the non-responders. Here, 26 mutations were exclusively observed in responders, and 17/26 were exclusively observed in PR-high patients (Fig 5 and Table 3). Nevertheless, we did not observe any significant difference between mutational status and successful R0 resection of colorectal cancer liver metastasis (Table 4).

**Table 2:** Mutations in Treatment Responders and Non-Responders.

Gene	Responders (n = 21)	Non responders (n = 6)	No response data available (n = 2)
*HNF1A*	7 (33.3%)	2 (33.3%)	0
*FGFR2*	6 (28.6%)	2 (33.3%)	1
***VHL***	7 (33.3%)	0 (0%)	1
*PDGFR*	5 (23.8%)	3 (50%)	0
***ERBB2***	6 (28.6%)	0 (0%)	1
*ABL1*	5 (23.8%)	1 (16.6%)	1
*SMO*	5 (23.8%)	1 (16.6%)	1
***GNA11***	4 (19%)	0 (0%)	2
*RET*	4 (19%)	1 (16.6%)	1
*STK11*	4 (19%)	1 (16.6%)	1
*HRAS*	3 (14.2%)	1 (16.6%)	2

*Responders were defined as >30% tumor shrinkage based on Response Evaluation Criteria In Solid Tumors (RECIST). VHL, ERBB2 and GNA11 are marked as they were detected more frequent in responders. For full gene names please see the table in the methods section.*

**Table 3:** Overlapping and Unique Mutations in Different Treatment Response Groups.

Response Groups	Total	Mutations
PR- high, PR-low and SD	17	*NRAS, KRAS, NOTCH1, SMO, PDGFRA, BXW7, ABL1, PIK3CA, HRAS, TP53, PTEN, RET, HNF1A, FGFR2, APC, SMAD4, STK11*
PR-high and SD	2	*FLT3, PTPN11*
PR- high and PR-low	9	*RB1, KIT, FGFR3, VHL, EGFR, GNA11, MLH1, ATM, ERBB2*
PR-high	17	*EGFR, EGFR-AS1, GNAS, ERBB4, CDH1, CTNNB1, BRAF, AKT1, SMARCB1, FGFR1, MET, MPL, GNAQ, CSF1R, KDR, ALK, JAK3, IDH1*

*The presence of different mutations in the illustrated treatment response groups was evaluated and illustrated in a Venn Diagramm (Graphical illustration, please see* Fig 4*). Corresponding mutations in the respective overlapping groups can be found in this table Abbreviations: PR-High = Partial Response-High (>50% tumor shrinkage according to RECIST), PR-Low = Partial Response-Low (>30% < 50% tumor shrinkage according to RECIST), SD = Stable Disease. For full gene names, please see the table in the methods section.*

**Table 4:** Mutations and resection status of colorectal cancer liver metastasis.

Gene	Liver R0 resected (n = 16)	Non R0 resected (n = 13)	Significance
*HNF1A*	4 (25%)	5 (38.45%)	ns
*FGFR2*	6 (37.5%)	3 (23.08%)	ns
*VHL*	5 (31.25%)	3 (23.08%)	ns
*PDGFR*	5 (31.25%)	3 (23.08%)	ns
*ERBB2*	5 (31.25%)	2 (15.37%)	ns
*ABL1*	4 (25%)	3 (23.08%)	ns
*SMO*	5 (31.25%)	2 (15.37%)	ns
*GNA11*	3 (18.75%)	3 (23.08%)	ns
*RET*	4 (25%)	2 (15.37%)	ns
*STK11*	4 (25%)	2 (15.37%)	ns
*HRAS*	3 (18.75%)	3 (23.08%)	ns

*Patients were grouped based on the pathological resection status und colorectal cancer liver metastasis in R0 resected versus no R0 resection was achieved. Absolute patient counts and frequency within the group is illustrated for several mutations. For full gene names, please see the table in the methods section.*

Finally, to see if some of the mutations were related to previous treatment with chemotherapy and anti-EGFR monoclonal antibodies, we investigated whether some mutations were more frequent in the post-treatment samples (Table 5). *FGFR2* mutations were significantly more often observed in post-treatment samples compared to pre-treatment samples (p = 0.0168, 9/20 vs. 0/9). Numerically, mutations in *VHL* (+24%), *ERBB2* (+19%), *SMO* (+19%), *GNA11* (+19%), *RET* (+19%), *STK11*(+19%) and *HRAS* (+19%) were also more frequent in post-treatment cohort, although lacking statistical significance (Table 5).

**Table 5:** Mutations in Pre-treatment versus Post-Treatment samples.

Gene	Pre-treatment count (AF > 3%, N = 9)	Post-treatment count (AF > 3%, N = 20))	p-value
*HNF1A*	4 (44.4%)	5 (25%)	ns
***FGFR2***	0 (0%)	9 (45%)	p = 0.0168
*VHL*	1 (11%)	7 (35%)	ns
*PDGFR*	3 (33.3%)	5 (25%)	ns
*ERBB2*	1 (11%)	6 (30%)	ns
*ABL1*	2 (22%)	5 (25%)	ns
*SMO*	1 (11%)	6 (30%)	ns
*GNA11*	1 (11%)	5 (25%)	ns
*RET*	1 (11%)	5 (25%)	ns
*STK11*	1 (11%)	5 (25%)	ns
*HRAS*	1 (11%)	5 (25%)	ns

*For full gene names please see the table in the methods section.*

## Discussion

Despite advances in multimodal therapy of microsatellite stable (MSS) stage IV colorectal cancer, the overall prognosis is still dismal [1,2]. The current standard of care is a combinatorial treatment approach consisting of chemotherapy (mostly FOLFOX or FOLFIRI doublet regimens) and monoclonal antibodies (targeting VEGF(R) or EGFR pathway) [4–6]. Anti-EGFR targeted therapy is active but acquired secondary therapy resistance usually occurs within 8–10 months [26–28]. Initially, resistance to anti-EGFR monotherapy has been explained by intrinsic mechanisms of resistance, mostly by mutations affecting the RAS/RAF/MEK and PI3K/AKT/mTOR pathway [29–31]. In recent years, additional genetic and non-genetic negative predictors of anti-EGFR therapy response have been discussed [32].

Therefore, in this single-center retrospective study, a total of 29 macrodissected tumor samples from patients with primarily unresectable synchronous or metachronous colorectal liver metastases who received neoadjuvant chemotherapy in combination with either cetuximab or panitumumab as a conversion strategy to surgical resection were analyzed with a targeted NGS panel. NGS confirmed prior identified *KRAS* mutations in 4/29 patient samples and revealed further somatic clonal and subclonal *RAS* alterations in five post-treatment samples. Noteworthy, the presence of acquired *RAS* alterations did not influence tumor response or surgical resection success. Further, we identified recurrent (clonal and subclonal) mutations in responding tumors with higher frequencies compared to published datasets of primary and metastatic CRC (Fig 3). Especially, mutations in *VHL, ERBB2* and *SMO* were exclusively observed in well-responding patients, although the results lack statistical significance due to the small sample size and remain therefore descriptive. Further, mutations in *FGFR2* were significantly associated with post-anti-EGFR treatment samples, indicating a higher frequency of this mutation as a response to treatment, although due to selection bias this remains speculative. Importantly, none of the mutations were predictive for successful R0 resection; therefore, no prediction for routine clinical decision making in predicting successful conversion therapy was possible based on genetic features within our cohort.

Although we cannot directly link single nucleotide variants to therapy resistance, the higher frequency of subclonal variants, which are also described to be involved in anti-EGFR antibody resistance in literature [33], in *ERBB2* [34]*, ERBB4* [35]*, FGFR1–3* [36–38], *EGFR* [15,39] itself and *PDGFR* [40] with the potential downstream activation of ERK in responders and/or post treatment samples is indicating an association between therapy and alternative activation of MAPK signaling pathway in this tumor cell clones, especially as they are way less frequently reported in the large MSK and TCGA cohorts. In line, many of the observed alterations, based on the existing clinical and preclinical evidence of their role in anti-EGFR resistance, were included in the panels for negative hyperselection (i.e., *PTEN* [41], *MET* [34] and *ALK* [42]) of patients, which is suggested for identification of patients without mutations indicating anti-EGFR therapy resistance in circulating tumor DNA for treatment with anti-EGFR combination regimens [43].

To the best of our knowledge, single nucleotide variants of the von Hippel-Lindau (VHL) gene have not yet been implicated in resistance to anti-EGFR antibody treatment, although they have been described in subpopulations of primary CRC [44]. Consequently, they may represent a compelling target in this patient population, necessitating further research.

Historically, secondary mutations in the mitogen-activated protein (MAPK) pathway have been the primary factor in anti-EGFR therapy resistance in colorectal cancer or lung cancer [14,15]. Further, the development of treatment resistance has been mostly based on the model of therapy-resistant subclonal populations that ultimately outgrow and outnumber treatment-sensitive cells [14]. However, a recent study showed that this clonal progression of MAPK pathway mutations was present in 46% of patients with acquired resistance to anti-EGFR monotherapy (combination of third line clinical trials) compared to as few as 9.1% of patients treated with chemotherapy plus anti-EGFR antibodies in comparison to 5.7% in patient treated with chemotherapy alone [45], thus challenging the relevance of genetic resistance for patients treated in the first therapy line with combinatorial treatment approaches. Accordingly, we did not observe any effect of the observed mutations in the RAS pathway or with the potential to bypass EGFR signaling on treatment response or successful R0 resection. Further, it remains questionable if the subclonal mutations observed in this study are relevant for later therapy resistance or if they explain the survival of clones harboring those alterations. In line, a previous study reported that subclonal mutations rarely become clonal upon progression [45]. Several groups recently reported that transcriptomic mechanisms of resistance mediate cross therapy resistance, i.e., by epithelial-to-mesenchymal transition (EMT) to chemotherapy and anti-EGFR monoclonal antibodies, thereby being the major driver of resistance in this patient subgroup [45–47]. Therefore, the role of non-genetic resistance mechanisms is highlighted by the absence of relevant therapy resistance/response prediction in the patients with acquired *RAS* pathway alterations in our cohort.

Our study has several limitations. First, and most importantly, the small sample size and retrospective design limit the statistical power of the analysis. Consequently, the observed mutational patterns should be interpreted as descriptive and hypothesis-generating rather than confirmatory. Second, with the advances in whole-exome and whole-genome sequencing, the used NGS panel appears rather small, potentially missing additional interesting genomic alterations. As pre-treatment and post-treatment samples were derived from different patients, direct longitudinal comparisons were not possible. Therefore, the observed differences in mutational frequencies cannot be conclusively attributed to treatment exposure and may partly reflect inter-cohort biological variability. These findings should thus be interpreted as descriptive and hypothesis-generating. Last, this study is subject to selection bias due to the highly specific composition of the cohort. Only patients who were eligible for curative-intent liver surgery were included, either because of primary oligometastatic disease or due to a particularly favorable response to anti-EGFR-based conversion chemotherapy. Consequently, this population represents a biologically and clinically distinct subgroup of metastatic colorectal cancer patients with relatively favorable disease characteristics. Our findings may therefore not be generalizable to the broader metastatic colorectal cancer population, particularly to patients with unresectable or more aggressive disease. Nevertheless, this study contributes valuable insides into CRC behavior upon treatment with chemotherapy and anti-EGFR antibodies. Future studies in larger and more heterogeneous cohorts are required to validate these observations.

In conclusion, the presented study adds important data on clonal and subclonal mutations implicated in resistance to anti-EGFR monoclonal antibodies in patients with colorectal cancer liver metastasis successfully treated in the first line with chemotherapy and anti-EGFR antibodies. Additionally, the data highlight that no relevant conclusions can be drawn from genomic data for predicting clinical responses in this patient subgroup, and that the currently established molecular markers are sufficient for making decisions about first-line therapy. Therefore, further research focusing on non-genetic mechanisms of resistance, ideally using matched pre- and post-treatment samples, is urgently needed to enable more mCRC patients to undergo surgical resection with curative intention after neoadjuvant treatment and to identify prognostic markers for neoadjuvant treatment response.

## Materials and methods

### Patients

The retrospective analysis was approved by the local Ethics Committee of the University Hospital Frankfurt (protocol number 4/09) in February 2012. An amendment to include genomic analyses was subsequently approved on May 12, 2021. Patients treated from January 1, 2008, until February 6, 2014, were retrospectively identified from the hospital database (ORBIS nice, Agfa HealthCare GmbH, Bonn, Germany) as previously published [25]. Patient data were accessed on 25/08/2016. Patient data were fully anonymized before accessed for this study. Inclusion criteria were predominant, primarily unresectable synchronous or metachronous colorectal liver metastases who received neoadjuvant chemotherapy as conversion strategy including either cetuximab or panitumumab, regardless of the concomitant chemotherapy. Patients with uncertain changes (either based on small size (<1 cm) or untypical radiographic appearance) in other organs, for example, lung and bone, were also included. Resectability was evaluated every 2–3 months by CT or MRI scan and discussed in an interdisciplinary conference (consisting of surgeons, oncologists, radiologists, gastroenterologists). If the patients was not found suitable for surgery, chemotherapy was continued and the evaluation repeated after 2–3 months. All patients were KRAS wild-type according to the patient’s records. KRAS testing had been routinely performed by the pathologist. Resection status (R0, R1, or R2) of the liver metastases was judged by the pathologist according to standard criteria.

### Depth of response

For evaluation of depth of response, the size of the liver metastases at the time of diagnosis was measured by an expert radiologist and the sum of the diameters was calculated. At the time of response evaluation, the sum of the diameters was calculated again and the change in size was calculated as percentage in comparison to the size at base line. Only patients with evaluable CT or MRI scans were included in the analysis; therefore, data from 27 of 29 patients were included in the analysis.

### Sample preparation

Hematoxylin and eosin (H&E) stained slides were reviewed by an expert pathologist (R.W.) to determine the content of neoplastic cells. Representative FFPE blocks of tumor tissue were selected. To enhance tumor content of surgical tumor specimens, macrodissection was applied as appropriate. DNA of surgical specimens was extracted with GeneRead DNA FFPE Kit (Qiagen, Hilden, Germany) according to manufacturer’s recommendations. Purified DNA was quantified with Quantus Fluorometer (Promega, Madison, WI).

### Targeted next generation sequencing

Sequencing libraries were prepared from tumor and non-tumor tissue with TruSeq® Amplicon – Cancer Panel (Illumina, San Diego, CA) according to the manufacturer’s instructions and paired-end sequencing was performed on a MiSeq (Illumina, San Diego, CA) with 2 × 150 base pairs (bp) read length.

**Table:** 

Gene Symbol	Full Gene Name
*ABL1*	ABL Proto-Oncogene 1, Non-Receptor Tyrosine Kinase
*AKT1*	AKT Serine/Threonine Kinase 1
*ALK*	ALK Receptor Tyrosine Kinase
*APC*	APC Regulator of WNT Signaling Pathway
*ATM*	ATM Serine/Threonine Kinase
*BRAF*	B-Raf Proto-Oncogene, Serine/Threonine Kinase
*CDH1*	Cadherin 1
*CDKN2A*	Cyclin Dependent Kinase Inhibitor 2A
*CSF1R*	Colony Stimulating Factor 1 Receptor
*CTNNB1*	Catenin Beta 1
*EGFR*	Epidermal Growth Factor Receptor
*ERBB2*	Erb-B2 Receptor Tyrosine Kinase 2 (HER2)
*ERBB4*	Erb-B2 Receptor Tyrosine Kinase 4
*FBXW7*	F-Box and WD Repeat Domain Containing 7
*FGFR1*	Fibroblast Growth Factor Receptor 1
*FGFR2*	Fibroblast Growth Factor Receptor 2
*FGFR3*	Fibroblast Growth Factor Receptor 3
*FLT3*	Fms Related Receptor Tyrosine Kinase 3
*GNA11*	G Protein Subunit Alpha 11
*GNAQ*	G Protein Subunit Alpha Q
*GNAS*	GNAS Complex Locus
*HNF1A*	HNF1 Homeobox A
*HRAS*	HRas Proto-Oncogene, GTPase
*IDH1*	Isocitrate Dehydrogenase (NADP(+)) 1
*JAK2*	Janus Kinase 2
*JAK3*	Janus Kinase 3
*KDR*	Kinase Insert Domain Receptor (VEGFR2)
*KIT*	KIT Proto-Oncogene, Receptor Tyrosine Kinase
*KRAS*	KRAS Proto-Oncogene, GTPase
*MET*	MET Proto-Oncogene, Receptor Tyrosine Kinase
*MLH1*	MutL Homolog 1
*MPL*	MPL Proto-Oncogene, Thrombopoietin Receptor
*NOTCH1*	Notch Receptor 1
*NPM1*	Nucleophosmin 1
*NRAS*	NRAS Proto-Oncogene, GTPase
*PDGFRA*	Platelet Derived Growth Factor Receptor Alpha
*PIK3CA*	Phosphatidylinositol-4,5-Bisphosphate 3-Kinase Catalytic Subunit Alpha
*PTEN*	Phosphatase and Tensin Homolog
*PTPN11*	Protein Tyrosine Phosphatase Non-Receptor Type 11
*RB1*	RB Transcriptional Corepressor 1
*RET*	Ret Proto-Oncogene
*SMAD4*	SMAD Family Member 4
*SMARCB1*	SWI/SNF Related, Matrix Associated, Actin Dependent Regulator of Chromatin, Subfamily B, Member 1
*SMO*	Smoothened, Frizzled Class Receptor
*SRC*	SRC Proto-Oncogene, Non-Receptor Tyrosine Kinase
*STK11*	Serine/Threonine Kinase 11
*TP53*	Tumor Protein P53
*VHL*	Von Hippel-Lindau Tumor Suppressor

### Variant calling and analysis

Variant calling was applied according to manufacturer´s instruction using internal MiSeq´s variant calling via BaseSpace (Illumina, San Diego, CA). For further secondary analysis Illumina Variant studio 2.2 was used. The following cut-offs were used for detection of somatic single nucleotide variants (SNV): mapping quality ≥ 20, variant allele frequency in the tumor ≥ 3%, general sequence depth ≥ 10, tumor variant sequence depth ≥ 2, no potential indel within 5 bp of the suspected SNV, not more than 2 SNVs in any 10-bp window. Additionally, all mutations were confirmed by inspection of the sequencing data in the genome browser and all SNVs with suspicion for sequencing or alignment error were removed. Variants were compared to the 1000 genomes project data base and common polymorphisms were excluded. Only SNVs in exons or splice sites were further analyzed.

### Mutation frequencies publicly available colorectal cancer datasets

Analysis of the MSK-IMPACT Clinical Sequencing Cohort was performed using C-Bioportal [48–50] filtering the Colorectal Cancer (MSK, JNCI 2021 [51]) datasets with the following filter: Sample Type: Metastasis, Metastatic site: Liver, Gene Panel: IMPACT410, Cancer Type Detailed: Colorectal Adenocarcinoma, Rectal Adenocarcinoma, Colon Adenocarcinoma (n = 242). Data were exported (see Supplementary Data 1) and mutation frequency of genes which were also detected within our study were used as a comparison in Fig 3.

The Cancer Genome Atlas- Colon Adenocarcinoma cohort (TCGA-COAD n = 461) was assed using the Genomic Data Commons (GDC) Data Portal [52]. Gene frequencies of Genes investigated in our study were assed and data exported (see Supplementary Data 1). Mutation frequency were used as a comparison in Fig 3.

### Statistical tests

The gene panel was pre-specified based on manufacturers selection to include well-established, clinically relevant tumor-associated mutations. However, no further pre-specification of individual genes or mutations for outcome analyses was performed; therefore, all association analyses should be considered exploratory.

Calculation of statistical significance was done using Graphpad Prism 10 applying one-sided Fisher Exact Test with the hypothesis, that the observed mutation is more frequent in the well responding patients/the R0 resected patients or the post-treatment samples. The sample number (=n) is indicated in the graph or in the legends. p < 0.05 was considered significant and exact p-values are displayed in the tables. All measurements were taken from distinct samples, no samples were measured repeatedly to generate data.

## Supporting information

S1 File Data. (XLSX)
